# Biopsy-free circulating tumor DNA assay identifies actionable mutations in lung cancer

**DOI:** 10.18632/oncotarget.11801

**Published:** 2016-09-01

**Authors:** Victoria Villaflor, Brian Won, Rebecca Nagy, Kimberly Banks, Richard B. Lanman, AmirAli Talasaz, Ravi Salgia

**Affiliations:** ^1^ Northwestern University Feinberg School of Medicine, Chicago, IL 60611, USA; ^2^ University of Chicago School of Medicine, MC 2115, Chicago, IL 60637, USA; ^3^ Guardant Health, Redwood City, CA 94063, USA; ^4^ City of Hope, Duarte, CA 91010, USA

**Keywords:** circulating tumor DNA, cell-free DNA, non-small cell lung cancer, next-generation sequencing

## Abstract

**Introduction:**

The potential of oncogene-driven targeted therapy is perhaps most fully realized in non-small cell lung cancer (NSCLC), given the number of genomic targets and approved matched therapies. However, invasive tissue biopsy at the time of each disease progression may not be possible and is associated with high morbidity and cost. Use of newly available “liquid biopsies” can circumvent these issues.

**Results:**

83% of subjects had at least one genomic alteration identified in plasma. Most commonly mutated genes were *TP53*, *KRAS* and *EGFR*. Subjects with no detectable ctDNA were more likely to have small volume disease, lepidic growth pattern, mucinous tumors or isolated leptomeningeal disease.

**Methods:**

Subjects were individuals with NSCLC undergoing analysis of cell-free circulating tumor DNA using a validated, commercially-available next-generation sequencing assay at a single institution. Demographic, clinicopathologic information and results from tissue and plasma-based genomic testing were reviewed for each subject.

**Conclusions:**

This is the first clinic-based series of NSCLC patients assessing outcomes of targeted therapies using a commercially available ctDNA assay. Over 80% of patients had detectable ctDNA, concordance between paired tissue and blood for truncal oncogenic drivers was high and patients with biomarkers identified in plasma had PFS in the expected range. These data suggest that biopsy-free ctDNA analysis is a viable first choice when the diagnostic tissue biopsy is insufficient for genotyping or at the time of progression when a repeated invasive tissue biopsy is not possible/preferred.

## INTRODUCTION

Genomic profiling of tumor DNA to identify targetable oncogenic drivers is rapidly becoming a part of standard care for many different cancer types [1]. Targeted therapy of these mutations can result in dramatic and immediate responses and imparting median progression-free survival intervals 2–3 times longer than in patients receiving standard chemotherapy. Although the vast majority of these patients will recur or progress on treatment, the availability of 2nd and 3rd generation targeted therapies for emerging resistance mutations in *EGFR* and *ALK* fusion driven lung cancer has prolonged intervals of disease-free survival [2, 3], leading to recommendations to repeat biopsies for genotyping lung cancer patients progressing after first line targeted therapies.

Non-small cell lung cancer (NSCLC) is a prototypical example of genotype-driven precision oncology, given the number of genomic targets and approved matched therapies [1]. Approximately 10–15% of NSCLC patients in North America carry activating mutations in the *EGFR* gene that impart sensitivity to several tyrosine kinase inhibitors (TKIs) [4, 5]. National Comprehensive Cancer Network (NCCN) guidelines now recommend multiplex testing or next-generation sequencing (NGS) to target additional genomic alterations including *ERBB2* (HER2) indels, *BRAF* mutations, *MET* exon skipping mutations and amplification and *ALK, RET,* and *ROS1* fusions [1]. Emerging genomic targets in NSCLC include alterations in *AXL, NTRK* and others [6–8] Furthermore, the convergent genomic evolution of lung cancer is relatively well characterized, which has allowed for the recent development of second and third generation targeted therapies to overcome acquired resistance [9].

Until recently, the only option for sequencing the tumor genome was through tissue biopsy. While a tissue biopsy is required to verify a cancer diagnosis and determine histology, there is often insufficient tissue for genotyping with expert centers reporting rates up to 25% [10–12], especially when a gene-by-gene sequential testing approach is utilized. Once tissue is exhausted, options include a repeat biopsy or more often treating the patient empirically with standard chemotherapy when the patient may have benefitted from targeted therapy. The problem of insufficient tissue for genotyping may be repeated when a repeat biopsy at the time of disease progression is performed to determine the mechanism of resistance and next steps for management [1]. An example of this in NSCLC is the identification of an *EGFR* activating mutation, which can be treated with first- and/or second-generation TKIs. Half of these patients will progress due to the development of the *EGFR* T790M mutation [13], which can be treated using new third generation *EGFR* TKIs. While this approach can extend survival it also leads to multiple invasive procedures over the course of the disease, which in turn leads to increased morbidity, mortality and cost [14]. One report using a 5% Medicare sample cited a median cost of biopsy of $4,157, but a mean cost of $14,587 due to the 19% complication rate [15] mostly attributed to pneumothorax.

Biopsy-free sampling of cell-free circulating tumor DNA (ctDNA) in advanced cancer with NGS is a highly sensitive and specific non-invasive means of tumor profiling [16–18]. The development of ctDNA assays and their recent implementation into clinical care may be a viable option in cases where tissue quantity is inadequate for genomic profiling or in patients who are unable to undergo repeat biopsy due to tumor location or precarious performance status. Detection of ctDNA in a patient's bloodstream depends on many factors including stage, tumor burden, cancer type and rate of cell turnover [17, 19, 20]. Tumors that have been stabilized by therapy undergo less apoptosis and necrosis and typically do not shed large amounts of DNA into the bloodstream [21]. This is also true for stage I-II cancers, where the tumors are not yet outgrowing their blood supply and may have lower cell turnover. In addition, tumors that are small in size and/or slow growing, e.g. neuroendocrine tumors like papillary thyroid cancer, may have levels of cell free DNA in the bloodstream that are below the level of detection for most assays [17]. Therefore, the clinical context during which ctDNA analysis is performed is critical to ensure the accurate interpretation of ctDNA test results.

The goals of this descriptive study were to evaluate a targeted ctDNA NGS gene panel in a prospective series of consented NSCLC cases from a single institution, determine the frequency and distribution of genomic alterations across cases as compared to tissue NGS results (when available), and characterize those cases in which ctDNA was undetectable in a clinical practice setting.

## RESULTS

### Subject characteristics

Demographic and clinical characteristics of the 68 subjects are shown in Table 1. The majority of patients had a diagnosis of lung adenocarcinoma (*n* = 55, 81%). There were slightly more African-American subjects (*n* = 36, 53%) than Caucasian subjects (*n* = 29, 43%). Seventeen patients (25%) were either stage I or II at the time of diagnosis. Of these early stage patients, 2 were newly diagnosed at the time of blood draw and 15 had experienced a loco-regional or distant recurrence and therefore were considered metastatic at the time of blood draw. The remaining 51 patents were either stage III (7%) or stage IV (68%) at the time of diagnosis and blood draw. The average age at diagnosis was 64 years (range = 16–91 years) and the average age at first blood draw was 67 years (range = 16–91 years).

**Table 1:** Patient demographic and clinical characteristics

Cancer type	68 (100%)
Lung adenocarcinoma	55 (81%)
Lung squamous cell carcinoma	12 (17.7%)
Other	1 (1.3%)
Gender	
Female	44 (65%)
Male	24 (35%)
Race	
Caucasian	29 (43%)
Black	36 (53%)
Other/unknown	3 (4%)
Stage at time of diagnosis	
I	9 (13%)
II	8 (12%)
III	5 (7%)
IV	46 (68%)
Average age (range)	64 yrs. (16–91 yrs.)

Clinical status at the time of blood draw for each patient is shown in Supplementary Table S1.

### ctDNA results

Of the 90 patients submitted for ctDNA analysis as part of clinical care, 68 had provided informed consent for inclusion in this study. There were 69 blood samples analyzed from these 68 patients; sixty-seven patients had one blood draw and 1 patient had two draws. Thirty-eight samples from the 68 subjects were tested using the 54-gene ctDNA panel while the remaining 31 samples were analyzed on the 68-gene ctDNA panel. Of note, the 54-gene panel did not include *ALK, RET* or *ROS1* fusions. Tissue-based testing was performed on 44 subjects using 9 different testing platforms. Three subjects had two different tissue tests performed on the same biopsy material.

**Figure 1:**
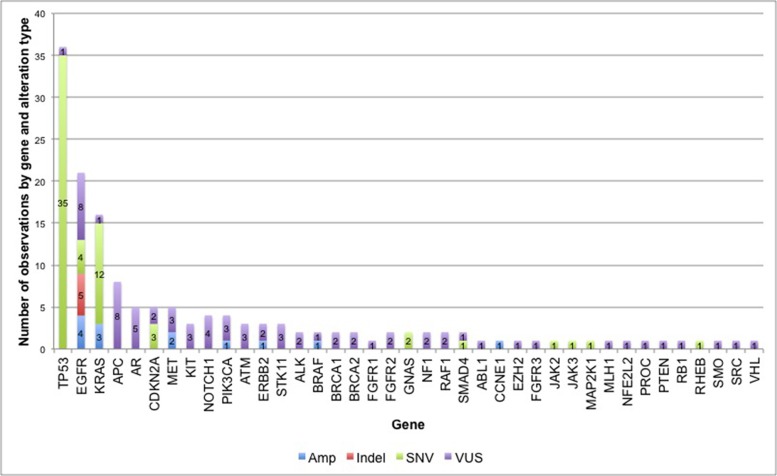
Frequency of non-synonymous alterations in 56 NSCLC patients In the 56 cases with at least one detectable ctDNA alteration, there were 164 alterations in 39 different genes, including 133 single nucleotide variants (SNVs), 13 amplifications (Amp), and 5 insertion/deletions (Indels). SNVs that were characterized as variants of uncertain significance (VUSs) are shown in purple.

**Figure 2:**
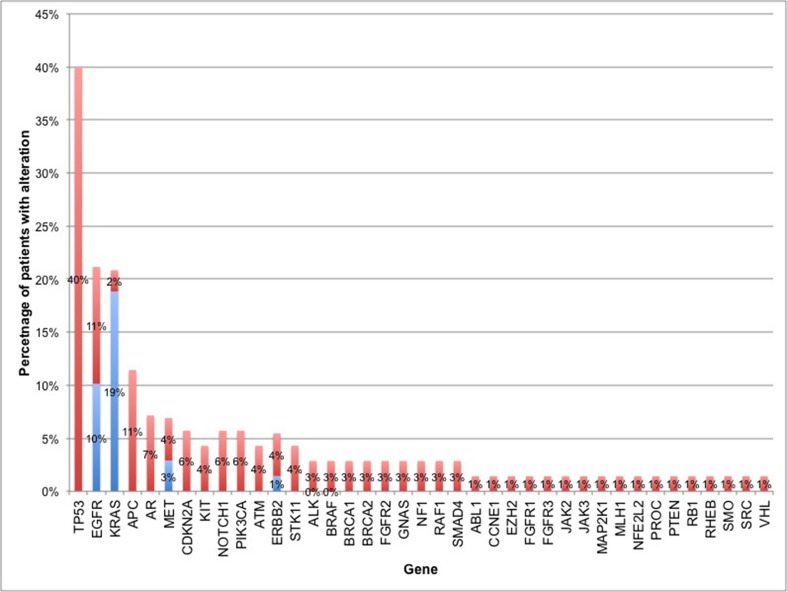
Percentage of patients with non-synonymous alterations by gene The percentage of patients with alterations in each of the 39 genes found to have at least 1 ctDNA alteration. For genes with truncal driver mutations (as defined by NCCN NSCLC guidelines), the percentage of driver mutations is shown in blue. Frequency of all other non-synonymous mutations (i.e. non-driver mutations in NSCLC biomarkers or mutations in other genes) is shown in red.

Eighty-three percent of subjects (56 of 68) had at least one non-synonymous ctDNA alteration detected. In the cohort of patients with at least one alteration detected in plasma, there were 164 alterations in 39 different genes, including 133 SNVs, 13 amplifications, and 5 insertion/deletions (Figure 1). As expected, mutations in *TP53, EGFR* and *KRAS* were most frequent (Figure 2). Twenty patients had one of the following biomarkers identified in plasma: *KRAS* activating mutations in codons 12, 13 or 61 (*n* = 12), *EGFR* activating mutations in exon 19 or 21 (*n* = 6) and *MET* amplification (*n* = 2). These mutations were mutually exclusive. Of note, two patients progressing on EGFR TKI therapy had *EGFR* T790M alterations detected in plasma but did not have a re-biopsy for comparison.

**Figure 3:**
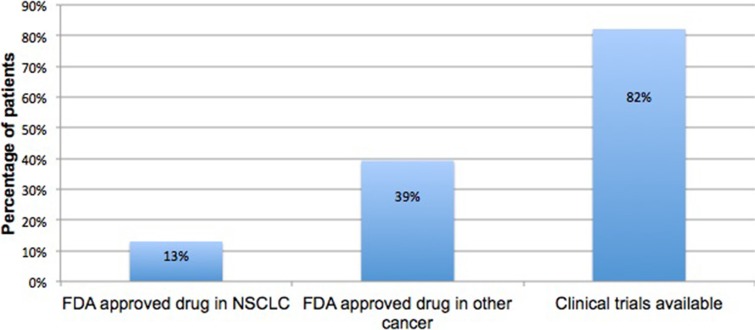
Actionability in ctDNA positive cases Frequency of patients whose ctDNA test identified a biomarker with an FDA approved targeted therapy in NSCLC, an FDA approved targeted therapy in a different indication, or a clinical trial.

Clinical actionability for the 56 patients with at least one ctDNA alteration detected is shown in Figure 3. FDA-approved therapies were available for 13% of patients found to carry activating mutations in the *EGFR* gene. Approximately 82% of patients had a matched clinical trial based on their diagnosis and/or genomic alteration and another 39% had a genomic alteration with an FDA approved therapeutic target in a different indication.

### Characteristics of subjects with no alterations detected

Of the 68 subjects with ctDNA results, 12 (17%) had no ctDNA alterations detected. Tissue-based results were available for 7 of these 12 individuals, as 3 cases did not have tissue analysis performed and 2 had no alterations detected in the tissue analysis. Of the 7 with tissue results, 5 patients had one of the following oncogenic driver mutations: *EGFR* S768I (1), *ALK* rearrangement (1), *MET* amplification (1), *KRAS* G12D (2). One patient with 2 serial blood draws had no alterations detected on either ctDNA test.

A detailed chart review was completed to determine possible clinical or biological explanations for a negative ctDNA test. Disease status at blood draw (locoregional disease, distant metastatic disease or newly diagnosed), histologic sub-type and features and therapy status (pre-treatment, on therapy, progressing) at the time of blood draw were reviewed for all cases (Table 2 and Supplementary Table S1). Of the 12 cases with no detectable ctDNA, 6 had small volume disease (total tumor burden < 1.5 cm) and 3 had a lepidic growth pattern, which is thought to be an indolent variant. Two had highly mucinous tumors, and 1 had isolated leptomeningeal disease. With regard to treatment response/status at the time of blood draw, 5 were stable and 2 were on active treatment.

**Table 2:** Clinical and pathologic characteristics of the 12 cases with no ctDNA alterations detected

Case number	Histology	Stage at Diagnosis	Location of tumor(s)	Small volume disease?	Other pathologic features	On therapy at time of blood draw?
1	Lung Adenocarcinoma	IIIB	Thoracic only	N	None	Y
5	NSCLC	IV	Thoracic only	N	Mucinous	N
6	NSCLC	IA	Thoracic only	Y	None	N
7	NSCLC	IV	Isolated leptomeningeal disease	N	None	N
8	NSCLC	IV	Thoracic only	N	None	Y
9	NSCLC	IIB	Thoracic + brain	Y	Lepidic	Y
10	NSCLC	IIA	Thoracic only	N	Lepidic	N
11	NSCLC	IVB	Bone	Y	None	N
61	NSCLC	IV	Thoracic only	Y	Lepidic	N
70	Lung Squamous Cell Carcinoma	IA	Thoracic only	Y	None	N
73	Lung Adenocarcinoma	IV	Thoracic only	N	Lepidic, mucinous	N
82	Lung Adenocarcinoma	IV	Thoracic only	Y	None	N

NSCLC, non-small cell lung cancer.

### Concordance between tissue and blood for truncal oncogenic drivers

**Figure 4:**
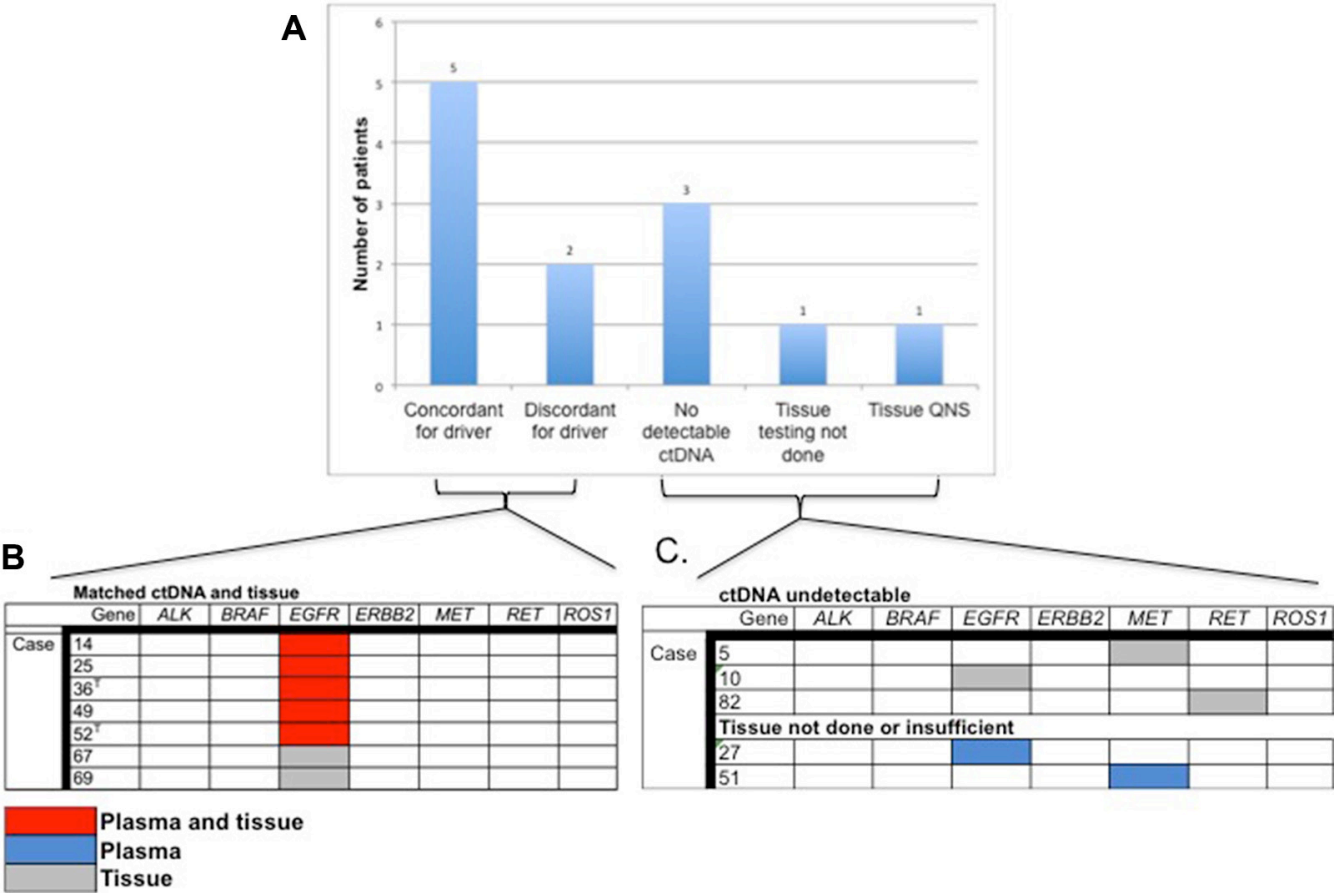
Plasma tissue concordance in cases with actionable NSCLC biomarkers (**A**) Rate of concordance between tissue and plasma for cases with an actionable NSCLC driver mutation and number of patients with actionable mutations for whom ctDNA was undetectable or tissue testing was either not done or was insufficient for analysis. (**B**) ctDNA tissue concordance in 7 paired tissue/plasma samples with actionable NSCLC driver mutations. T = Concurrent T790M mutation identified. (**C**) Identification of actionable driver mutations in cases with undetectable ctDNA or no tissue analysis/insufficient tissue.

Thirty-one patients had matched tissue and blood samples. The reason for lack of tissue results for the remaining 37 patients was not routinely documented in the patient chart but possible explanations include insufficient tissue for genomic analysis, difficult tumor to access via biopsy, poor performance status or patient preference. The time between biopsy and blood draw ranged from 0 days to 7 years, with an average of 8.8 months and median of 1.4 years between biopsy and blood draw. Twenty-eight samples from 27 subjects had an oncogenic driver detected in tissue or blood or both. The following driver mutations were identified in 23 cases: *KRAS* activating mutations (*n* = 14) and *EGFR* activating mutations (*n* = 9). These mutations were mutually exclusive whether found in tissue or in blood. Figure 4A shows plasma-tissue concordance for driver mutations with an FDA approved therapy in lung cancer or an FDA approved therapy in another indication. In cases with detectable ctDNA and completed tissue analysis, an *EGFR* activating was found in both tissue and blood in 5 paired samples, and in tissue only in 2 samples (71% concordance). Of note, in two cases with an *EGFR* exon 19 deletion identified in the original tissue biopsy, ctDNA analysis at the time of progression confirmed the *EGFR* indel and also identified a T790M mutation, conferring resistance to first-generation TKI (Figure 4B). One subject had an *ALK* fusion identified in tissue but this alteration was not part of the ctDNA panel at the time of the blood draw and therefore was not included in the concordance analysis. There were no alterations identified in *BRAF, MET, RET* or *ROS1* in these 8 samples. There was no correlation between concordance and timing of blood draw vs. tissue biopsy.

An additional twenty-three subjects had ctDNA analysis but no tissue-based genomic testing. In these 23 subjects there were 7 *KRAS* mutations, 2 *EGFR* activating mutations, and one activating *MET* mutation identified. Of the 12 cases with no detectable ctDNA alterations, three had a driver mutation identified in tissue (1 in *EGFR* indel, 1 *RET* fusion and 1 *MET* amplification, Figure 4B). Similarly, there were two cases for which tissue biopsy was not possible/was insufficient for testing and both had an actionable driver mutation detected in plasma: an *EGFR* indel and a *MET* amplification (Figure 4C).

Interestingly, over half (5/9, 55.5%) of the EGFR-positive cases were of African American ancestry. This represents a 17% (5/29) *EGFR* mutation frequency in those subjects of African American ancestry. Two of 36 Caucasian subjects (5%) and 2/2 (100%) of Asian subjects were *EGFR* driver positive in blood, tissue or both.

### Progression-free survival in EGFR mutation positive patients

A total of 9 subjects with paired tissue and blood samples had an *EGFR* driver mutation identified in plasma and tissue (*n* = 5), plasma only (*n* = 1) or tissue only (*n* = 3). Eight of these individuals were treated with erlotinib or afatinib at first or second line. Two patients were still responding to therapy at the time of data analysis. Of the 6 remaining patients, the median progression-free survival was 11.5 months (range 7.5 months–29 months; 95% CI–5.7–28.7).

## DISCUSSION

The majority of cases reported in this series (83%) had at least one detectable mutation in plasma, which is consistent with previous reports [16, 17]. The three most commonly altered genes were *TP53* (40% of patients), *EGFR* and *KRAS*. *EGFR* and *KRAS* activating mutations were present in 10% and 19% of patients, respectively while *ERBB2* and *MET* amplification were present in 3% and 1% of patients. These frequencies are in line with previous reports [22–26]. The frequency of *ALK, RET*, and *ROS1* fusions was lower than previously reported [22, 27–29], as was the rate of *BRAF* activating mutations. Half of the subjects in the series were not assessed for *ALK, RET* or *ROS1* fusions, as fusions were not included on the 54-gene ctDNA panel. There were no *BRAF* activating mutations identified in tissue. The lower *BRAF* rates could be due to the small number of cases and the relative rarity of these mutations in NSCLC.

There is a paucity of data on the concordance between plasma ctDNA and tissue-based genomic testing and this study is unique in that it is a clinically ascertained cohort of NSCLC patients. In one study of 42 advanced-stage NSCLC patients, concordance for mutations in *EGFR, KRAS, PIK3CA* and *TP53* was measured using a targeted sequencing approach. Concordance between tissue and plasma was 76% [30]. Another study compared the results of a hotspot *EGFR* T790M assay performed on matched tissue samples and plasma-derived ctDNA in 40 NSCLC patients with *EGFR*-mutant lung tumors who were progressing on *EGFR* TKI therapy. T790M genotypes were successfully obtained in 71% of tumor biopsies and 80% of ctDNA samples. As expected, approximately half of the patients carried a T790M mutation and concordance between tissue and ctDNA was 74%. T790M mutations were identified in 35% of patients for whom a tissue biopsy was negative or uninformative [12]. Lastly, a multicenter prospective study utilizing the same ctDNA assay [16] employed in the current study in pre-treated advanced cancers, concordance between 165 paired tissue and plasma ctDNA was measured for 11 genes across a variety of tumor types, including NSCLC. When tissue served as the reference standard, clinical sensitivity of the ctDNA assay was 85%, with > 99% clinical specificity and diagnostic accuracy. When ctDNA was considered the reference standard, clinical sensitivity of tissue-based NGS was 81%, with a clinical specificity of > 99% and diagnostic accuracy of 99.3%. In all three of these studies, tissue and blood sampling were performed concurrently at diagnosis or at progression and prior to next line therapy. Additional concordance analyses in this series were limited by the wide variety of tissue genomic testing panels performed, ranging from a single mutation companion diagnostic test to NGS-based panels of over 300 genes.

This is the first study to assess factors influencing ctDNA detection rates in NSCLC patients in clinical practice. The amount of ctDNA in circulation depends on many clinical factors, including but not limited to tumor type and location, clinical stage, tumor burden and rate of necrosis and apoptosis. In general, the ability to detect ctDNA correlates with stage [17, 18]. Detection rates for stage I/II cases range between 40–55% compared to 85–90% in those with advanced stage tumors [16, 17]. Levels of ctDNA may also vary by primary tumor site, with the highest levels being reported in lung cancer and GI cancers and lowest in primary brain tumors and more indolent cancers such as differentiated thyroid cancer [17]. In addition to these clinical factors, the timing of the blood draw is a critical factor to consider with ctDNA testing, as this can greatly influence the levels of ctDNA present in the bloodstream. Several studies have assessed ctDNA levels prior to and immediately following surgical resection. ctDNA levels often spike immediately after surgery and then drop precipitously 2–4 weeks post-operatively [31, 32]. These studies have also found a correlation between extent of surgical resection and post-operative ctDNA levels and longer disease-free survival in cases with no detectable post-operative ctDNA versus those with detectable post-operative ctDNA [32, 33]. Although direct correlations between absolute tumor burden and ctDNA levels were not possible, lower tumor burden correlated with lower or undetectable ctDNA levels. Similarly, ctDNA levels correlate with clinical response in patients undergoing chemotherapy or matched targeted therapy, with levels spiking in responders in the first 2–3 days, then dropping to lower or even undetectable levels in 2–3 weeks, and then rising again prior to and during radiographic progression and recurrence [18, 32, 34–36]. This so-called “molecular response” and “molecular progression” often precedes changes in protein serum markers and changes in imaging [34, 37]. Timing of the blood draw can also impact whether specific sub-clonal populations of tumor cells will be present in circulation. Therapy that stabilizes the tumor and suppresses cell turnover will often lead to the disappearance of those clones from circulation, which often re-appear upon progression or a change in therapy [18, 38]. Therefore, we recommend that future concordance studies of ctDNA to tissue biopsy based DNA genotyping be limited to pretreatment and concurrent samples or patients at progression at least two weeks after a cycle of chemotherapy, radiotherapy or targeted therapy.

In the current study, 12 patients had no ctDNA alterations detected. The majority of these patients had small volume disease isolated to the thoracic cavity or were not progressing. Interestingly 4 of the 12 cases had the bronchoalveolar subtype of adenocarcinoma, which is a more indolent form of the disease [39], and two had heavily mucinous tumors. Although the relationship between ctDNA and mucin levels has not been well studied, Bettegowda et al. reported an association between undetectable ctDNA and mucinous tumors in a series of metastatic colon cancer cases [17]. Lastly, one patient had isolated brain metastases, which is associated with lower ctDNA levels [17]. While the numbers in this series are modest and further research in larger prospective cohorts is needed, these data underscore the importance of considering the clinical context during which a ctDNA test is performed. Unlike tissue sampling, which is typically hindered by accessibility of the tumor and tumor heterogeneity, ctDNA analysis may be of limited value in patients with early stage disease, as well as advanced stage patients who have small volume disease, or who have stable disease that is not actively progressing, or those with indolent tumors. Additional research is needed to determine if factors such as location of residual disease (thoracic only versus distant metastatic sites) and mucinous sub-type are correlated with lower ctDNA levels.

ctDNA is just one of several tumor-derived biomarkers in circulation. Cancer exosomes and circulating tumor cells (CTCs) are also shed into circulation and like ctDNA, mirror the genomic make-up of the parental tumor cell from which they were derived [40]. Unlike ctDNA however, both CTCs and exosomes play a direct role in disease metastasis. Exosomes and a subset of CTCs have the ability to escape the primary tumor cell, invade a recipient cell, colonize and engraft to form a distant metastatic tumor site. CTCs have prognostic value in several cancers including metastatic breast cancer, uveal melanoma and others [41–44]. CTCs and exosomes can also serve as a source of tumor-derived DNA for genomic analysis, although the rarity of CTCs even in advanced cancers can make it difficult to capture a sufficient number of cells to perform wide-scale genomic sequencing. Dawson, et al. demonstrated that in metastatic breast cancer patients, ctDNA is significantly more abundant and more sensitive than CTCs in detecting malignancy [35, 43]. ctDNA also provided the earliest measure of response to treatment when compared to protein serum biomarkers and CTCs [35]. Exosomes are present at higher levels than CTCs and have been successfully used for targeted genotyping in several cancer types [45]. Of interest, exosomes carry not only DNA, but RNA, protein and other substances that can provide additional information about gene expression and the tumor microenvironment. However, exosomal capture methods are technically complex, at least at the current time. Despite some of these current challenges, “liquid biopsy' using ctDNA, CTCs, exosomes or a combination thereof, is likely to eclipse the use of repeat tissue biopsy in advanced cancer patients given the ease of access, decreased complications for patients, reduced cost, a shorter turn-around time from time of “biopsy” to results and their ability to offer information about disease progression and acquired resistance.

This is the first observational cohort series to confirm the clinical utility of ctDNA to guided targeted therapy in NSCLC outside of the investigational setting. The median PFS for subjects with *EGFR* activating mutations identified in plasma was similar to what has been previously reported in the literature [5, 46, 47]. In the present series, the treatment decision was generally made based on tissue analysis, so the analysis of ctDNA response rates and PFS are correlative, i.e. based on patients where ctDNA and tissue were concordant. A growing number of small patient series have measured clinical outcomes in patients treated on the basis of ctDNA-based biomarker identification. These studies suggest that progression free survival and response rates are equivalent regardless of the tumor DNA source (tissue vs. blood) used to identify the mutation [48–51]. Our findings build upon a single case report utilizing the same ctDNA assay of an *EGFR* T790M mutation detected at progression when repeat tissue biopsy was insufficient for NGS but responded to the third generation TKI osimertinib [52]. Another case series using the same ctDNA assay reported high response rates to targeted therapy of *ERBB2* (HER2) amplifications in metastatic breast cancer patients [53]. Taken together, these findings suggest that plasma-based NGS of ctDNA is a reliable and accurate surrogate for tissue NGS not only for determining the presence of tumor-derived mutations, but also for predicting response to therapy. Larger prospective series are needed to further address the latter.

In this study we demonstrated ctDNA detection rates of > 80% and a relatively high rate of concordance for actionable driver mutations in a series of NSCLC patients. These data suggest that biopsy-free ctDNA analysis is a viable first choice when the diagnostic tissue biopsy has been exhausted and tumor genotyping is not possible, or at the time of progression when an invasive tissue biopsy is not possible/preferred. The net health outcome of patients with advanced NSCLC may be increased if ctDNA identifies an actionable genomic target and avoids the potential complications of a repeat invasive tissue biopsy. When clinical suspicion of an actionable driver mutation is high but is not identified using ctDNA analysis, an invasive biopsy can be performed. It has been previously shown that tumor heterogeneity leads to limited sensitivity for tissue based genotyping [16, 54] relative to liquid biopsy, whereas the latter may act as a “summary” of cell turnover from multiple parts of a tumor or multiple tumors. Here we elucidate the reasons why ctDNA NGS may have limited sensitivity. Our hope is that when a liquid biopsy is ordered at progression and no actionable alterations are detected, this information will help clinicians decide when to supplement the liquid biopsy with an invasive tissue biopsy. This step-wise approach allows for avoidance of invasive and costly biopsies. However, when ctDNA testing is uninformative, a repeat tissue biopsy should be considered.

## MATERIALS AND METHODS

### Subjects

This study was approved by the Institutional Review Board at the University of Chicago. Subjects were males and females with a diagnosis of NSCLC who had undergone at least one ctDNA test at a single commercial ctDNA laboratory between September 2014 through August 2015. All subjects were seen at the University of Chicago and all provided written informed consent. Clinical characteristics of the subjects were extracted from the subject's electronic health record.

### Blood samples and cell-free DNA (cfDNA) isolation

Blood was collected in Streck™ tubes during routine phlebotomy, and samples were shipped at room temperature overnight. 10 mL of blood was processed upon receipt to isolate plasma by centrifugation at 1,600 g for 10 minutes at 4°C. Plasma was immediately aliquoted and stored at −70°C. cfDNA was extracted from 1mL aliquots of plasma using the QIAamp circulating nucleic acid kit (Qiagen), concentrated using Agencourt Ampure XP beads (Beckman Coulter), and quantified by Qubit fluorometer (Life Technologies, Carlsbad, CA, USA). All cfDNA sequencing and analysis was performed at Guardant Health (Redwood City, Calif, USA).

### Cell-free DNA sequencing

Barcoded sequencing libraries were generated from 5–30 ng of cfDNA. The majority of samples analyzed contained 25–30 ng of cfDNA. Two different clinical testing panels were utilized during the study period. For samples received in the laboratory before February 4th, 2015 (*n* = 38) a 54-gene panel was used. For samples received in the laboratory on or after February 4th, 2015 (*n* = 31) a 68-gene panel was employed. The genes and alterations interrogated by each of these panels are shown in Supplementary Figures S1 and S2.

Exons were captured using biotinylated custom bait oligonucleotides (Agilent), resulting in a 78,000 base-pair (78 kb) and 138,000 base-pair capture footprint on the 54 gene panel and 68 gene panel, respectively. Samples were paired-end sequenced on an Illumina Hi-Seq 2500, followed by algorithmic reconstruction of the digitized sequencing signals. The analytic and clinical validation of this assay is described elsewhere [16]. The coverage depth across all coding sequence in all samples averaged approximately 10,000x.

Illumina sequencing reads were mapped to the hg19/GRCh37 human reference sequence, and genomic alterations in cfDNA were identified from Illumina sequencing data by Guardant Health's proprietary bioinformatics algorithms. These algorithms quantify the absolute number of unique DNA fragments at a given nucleotide position, thereby enabling circulating tumor DNA to be measured as a quantitative percentage of total cfDNA. The mutant allele percentage for a given mutation was calculated as the fraction of cfDNA molecules harboring that mutation divided by the total number of unique cfDNA molecules mapping to the position of the mutation. The level of detection for single-nucleotide variants, indels and fusions in cfDNA by the Guardant360 assay is 0.1%.

### Genomic testing in tissue

Tissue NGS was performed at the discretion of the treating physician as part of clinical care at an academic medical center, using a variety of different approaches and assays, ranging from targeted multiplex testing of < 10 genes to tissue-based NGS of > 300 genes.

### Data analysis and tissue-blood concordance

Frequency estimates of mutations identified in ctDNA were calculated by gene and by patient. Only non-synonymous mutations were included in the frequency estimates. Concordance analyses in this series were limited to the following oncogenic drivers: *BRAF* activating mutations, *EGFR* indels, *ERBB2* indels, *MET* amplification and *MET* exon 14 skipping, and *ALK, RET, ROS1* fusions, as these all have either FDA approved therapies in NSCLC (e.g. erlotinib in EGFR indel positive patients) or FDA approved therapies in another cancer type (e.g. dabrafenib in *BRAF* positive patients). This approach was used to allow for direct comparisons between a single ctDNA assay, Guardant360™ (Guardant Health, Inc. Redwood City, CA) and 11 different tissue-based assays, which ranged from a single mutation companion diagnostic test to an NGS-based panel of over 300 genes. This allowed a maximum number of samples to be compared without having to control for limited breadth of the more targeted tissue-based assays and a focus on mutations with clinical and therapeutic significance.

Clinical actionability of ctDNA results, as reported on the clinical results report, was assessed for each subject. There were 3 possible levels of actionability; FDA approved targeted therapy in NSCLC, FDA approved targeted therapy available in a different cancer and clinical trial(s) available.

## SUPPLEMENTARY MATERIAL FIGURES AND TABLE




