# Supplementary material for: Biopsy-free circulating tumor DNA assay identifies actionable mutations in lung cancer
Source: Oncotarget. 2016 Sep 1;7(41):66880–91. doi: 10.18632/oncotarget.11801 (PMC5341844; doi:10.18632/oncotarget.11801)
Supplement: Supplementary file 1 [file oncotarget-07-66880-s001.pdf]

Biopsy-free circulating tumor DNA assay identifies actionable mutations in lung cancer

Supplementary Materials

All Exons and partial introns Covered (Copy Number Variations in Bold)

ALK	APC	AR	BRAF
CDKN2A	**EGFR**	**ERBB2**	FBXW7
KRAS	**MET**	MYC	NOTCH1
NRAS	PIK3CA	PTEN	PROC
RB1	TP53		

Partial Coverage ("Hot" Exons):

ABL1	AKT1	ATM	CDH1	CSF1R	CTNNB1
ERBB4	EZH2	FGFR1	FGFR2	FGFR3	FLT3
GNA11	GNAQ	GNAS	HNF1A	HRAS	IDH1
IDH2	JAK2	JAK3	KDR	KIT	MLH1
MPL	NPM1	PDGFRA	PTPN11	RET	SMAD4
SMARCB1	SMO	SRC	STK11	TERT	VHL

Supplementary Figure S1: Guardant Health 54-gene panel. One of two ctDNA testing panels utilized for this study. Sixty-nine samples from 68 patients were analyzed. Thirty-eight samples were analyzed using the 54-gene panel and 31 were analyzed using the 68-gene panel.

ALL EXONS AND PARTIAL INTRON COVERAGE

29 GENES
18 Copy Number Variations in Bold

APC	**AR**	ARID1A	**BRAF**	BRCA1
BRCA2	**CCND1**	**CCND2**	**CCNE1**	**CDK4**
**CDK6**	CDKN2A	CDKN2B	**EGFR**	**ERBB2**
**FGFR1**	**FGFR2**	HRAS	**KIT**	**KRAS**
**MET**	**MYC**	NF1	NRAS	**PDGFRA**
**PIK3CA**	PTEN	**RAF1**	TP53	

EXONS COVERED WITH REPORTED SOMATIC MUTATIONS

38 GENES

AKT1	ALK	ARAF	ATM	CDH1	CTNNB1	ESR1
EZH2	FBXW7	FGFR3	GATA3	GNA11	GNAQ	GNAS
HNF1A	IDH1	IDH2	JAK2	JAK3	MAP2K1	MAP2K2
MLH1	MPL	NFE2L2	NOTCH1	NPM1	PTPN11	RET
RHEB	RHOA	RIT1	ROS1	SMAD4	SMO	SRC
STK11	TERT*	VHL				

REARRANGEMENTS - EXONS and INTRONS WITH REPORTED BREAKPOINTS

ALK	RET	ROS1	NTRK1

4 GENES

*includes promoter region

Supplementary Figure S2: Guardant Health 68-gene panel. One of two ctDNA testing panels utilized for this study. Sixty-nine samples from 68 patients were analyzed. Thirty-eight samples were analyzed using the 54-gene panel and 31 were analyzed using the 68-gene panel.

Supplementary Table S1: Characteristics of the 68 patients including histologic sub-type, stage at initial diagnosis, disease status at blood draw, treatment status and response at blood draw and whether ctDNA alterations were detected. a = Patient 10 had two blood draws; b = small volume disease at blood draw; c = mucinous tumor; d = lepidic growth pattern; e = isolated leptomeningeal disease. See Supplementary_Table_S1
