# Supplementary material for: Biopsy-free circulating tumor DNA assay identifies actionable mutations in lung cancer
Source: Oncotarget. 2016 Sep 1;7(41):66880–91. doi: 10.18632/oncotarget.11801 (PMC5341844; doi:10.18632/oncotarget.11801)
Supplement: Supplementary file 2 [file oncotarget-07-66880-s002.docx]

| **Case number** | **Cancer type** | **Stage at Diagnosis** | **Disease status at blood draw** | **Treatment response at blood draw** | **ctDNA alterations detected?** |
| --- | --- | --- | --- | --- | --- |
| 1 | Lung Adenocarcinoma | IIIB | Locoregional disease | On therapy/status unknown | No |
| 5 | Lung Adenocarcinoma | IV | Newly diagnosed^c^ | Not on therapy | No |
| 6 | Lung Adenocarcinoma | IA | Locoregional disease^b^ | Stable | No |
| 7 | Lung Squamous Cell Carcinoma | IV | Distant mets^e^ | Progressing | No |
| 8 | Lung Adenocarcinoma | IV | Locoregional disease | On therapy/status unknown | No |
| 9 | Lung Adenocarcinoma | IIB | Distant mets^b,d^ | Progressing | No |
| 10^a^ | Lung Adenocarcinoma | IIA | Locoregional disease^d^ | Progressing | No |
| 11 | Lung Adenocarcinoma | IVB | Locoregional disease^b^ | Stable | No |
| 12 | Lung Adenocarcinoma | IV | Locoregional disease | Progressing | Yes |
| 13 | Lung Adenocarcinoma | IA | Distant mets | Progressing | Yes |
| 14 | Lung Adenocarcinoma | IV | Newly diagnosed | Not on therapy | Yes |
| 16 | Lung Adenocarcinoma | IV | Distant mets | Responding | Yes |
| 17 | Lung Adenocarcinoma | IV | Distant mets | Progressing | Yes |
| 18 | Lung Squamous Cell Carcinoma | IV | Locoregional disease | Progressing | Yes |
| 19 | Lung Adenocarcinoma | IV | Locoregional disease | On therapy/status unknown | Yes |
| 21 | Lung Adenocarcinoma | IV | Newly diagnosed | Not on therapy | Yes |
| 23 | Lung Adenocarcinoma | IIB | Locoregional disease | Progressing | Yes |
| 24 | Lung Adenocarcinoma | IV | Locoregional disease | Progressing | Yes |
| 25 | Lung Adenocarcinoma | IV | Locoregional disease | Progressing | Yes |
| 26 | Lung Adenocarcinoma | IVB | Newly diagnosed | Not on therapy | Yes |
| 27 | Lung Adenocarcinoma | IA | Distant mets | Progressing | Yes |
| 28 | Lung Adenocarcinoma | IV | Locoregional disease | Responding | Yes |
| 29 | Lung Squamous Cell Carcinoma | III | Locoregional disease | Progressing | Yes |
| 31 | Lung Adenocarcinoma | IIA | Distant mets | Progressing | Yes |
| 32 | Lung Squamous Cell Carcinoma | IIIB | Locoregional disease | Progressing | Yes |
| 33 | Lung Squamous Cell Carcinoma | IV | Locoregional disease | Progressing | Yes |
| 35 | Lung Adenocarcinoma | IV | Distant mets | Progressing | Yes |
| 36 | Lung Adenocarcinoma | I | Locoregional disease | Progressing | Yes |
| 37 | Lung Adenocarcinoma | IV | Locoregional disease | Progressing | Yes |
| 38 | Lung Adenocarcinoma | IV | Locoregional disease | Progressing | Yes |
| 39 | Lung Adenocarcinoma | IV | Newly diagnosed | Not on therapy | Yes |
| 40 | Lung Adenocarcinoma | IV | Distant mets | Progressing | Yes |
| 41 | Lung Adenocarcinoma | IV | Newly diagnosed | Not on therapy | Yes |
| 42 | Lung Adenocarcinoma | IIIA | Locoregional disease | Progressing | Yes |
| 43 | Lung Adenocarcinoma | IV | Locoregional disease | Progressing | Yes |
| 44 | Lung Squamous Cell Carcinoma | IIB | Locoregional disease | Progressing | Yes |
| 45 | Lung Adenocarcinoma | IV | Locoregional disease | Progressing | Yes |
| 46 | Lung Adenocarcinoma | IV | Newly diagnosed | Not on therapy | Yes |
| 49 | Lung Adenocarcinoma | IVA | Locoregional disease | Progressing | Yes |
| 50 | Lung Adenocarcinoma | IV | Distant mets | Progressing | Yes |
| 51 | Lung Squamous Cell Carcinoma | IV | Locoregional disease | Progressing | Yes |
| 52 | Lung Adenocarcinoma | IB | Locoregional disease | Progressing | Yes |
| 53 | Lung Adenocarcinoma | IIA | Newly diagnosed | Not on therapy | Yes |
| 55 | Lung Adenocarcinoma | IV | Distant mets | Progressing | Yes |
| 56 | Lung Adenocarcinoma | IV | Locoregional disease | Progressing | Yes |
| 57 | Lung Squamous Cell Carcinoma | IA | Distant mets | Progressing | Yes |
| 58 | Lung Adenocarcinoma | IV | Locoregional disease | Progressing | Yes |
| 59 | Lung Squamous Cell Carcinoma | IIA | Locoregional disease | Progressing | Yes |
| 60 | Lung Adenocarcinoma | IV | Locoregional disease | Progressing | Yes |
| 61 | Lung Adenocarcinoma | IV | Locoregional disease^b,d^ | Stable | No |
| 67 | Lung Adenocarcinoma | IIB | Newly diagnosed | Not on therapy | Yes |
| 68 | Lung Adenocarcinoma | IV | Locoregional disease | Progressing | Yes |
| 69 | Lung Adenocarcinoma | IV | Newly diagnosed | Not on therapy | Yes |
| 70 | Lung Squamous Cell Carcinoma | IA | Locoregional disease^b^ | Stable | No |
| 71 | NSCLC with Sarcomatoid Differentiation | IV | Newly diagnosed | Not on therapy | Yes |
| 72 | Lung Adenocarcinoma | IV | Newly diagnosed | Not on therapy | Yes |
| 73 | Lung Adenocarcinoma | IV | Newly diagnosed^c^ | Not on therapy | No |
| 74 | Lung Squamous Cell Carcinoma | III | Newly diagnosed | Not on therapy | Yes |
| 75 | Lung Adenocarcinoma | IV | Newly diagnosed | Not on therapy | Yes |
| 76 | Lung Adenocarcinoma | IV | Locoregional disease | On therapy/status unknown | Yes |
| 77 | Lung Adenocarcinoma | IA | Locoregional disease | Progressing | Yes |
| 78 | Lung Adenocarcinoma | IV | Newly diagnosed | Not on therapy | Yes |
| 79 | Lung Adenocarcinoma | IV | Newly diagnosed | Not on therapy | Yes |
| 80 | Lung Adenocarcinoma | IV | Newly diagnosed | Not on therapy | Yes |
| 81 | Lung Adenocarcinoma | IB | Distant mets | Progressing | Yes |
| 82 | Lung Adenocarcinoma | IV | Locoregional disease^b^ | Stable | No |
| 83 | Lung Adenocarcinoma | IV | Newly diagnosed | Not on therapy | Yes |
| 84 | Lung Squamous Cell Carcinoma | IV | Distant mets | Progressing | Yes |
